# Measures of self- and other-directed ageism and worries concerning COVID-19 health consequences: Results from a nationally representative sample of Israelis over the age of 50

**DOI:** 10.1371/journal.pone.0251577

**Published:** 2021-05-19

**Authors:** Liat Ayalon, Ella Cohn-Schwartz

**Affiliations:** 1 Louis and Gabi Weisfeld School of Social Work, Bar Ilan University, Ramat Gan, Israel; 2 Gerontology Program, School of Public Health, Ben Gurion University of the Negev, Beersheba, Israel; Universitat de Valencia, SPAIN

## Abstract

Worries associated with COVID-19 health consequences are well-justified. They may motivate people to take safety precautions, but may hinder if they become too intense. Current research has examined mainly age and gender as potential correlates associated with worries. This study instead, is focused on self-perceptions of aging (SPA) and perceived age discrimination as potential predictors of worry, in the light of the ageism pandemic which has co-occurred with the COVID-19 outbreak. The study is based on a national sample of 1,092 adults aged 50 and above in Israel. Phone interviews were conducted between March 29 2020 and May 3 2020, when Israel gradually moved from strict to partial lockdown. Respondents were queried about their worries related to COVID-19 health consequences, demographic characteristics, known-risk factors, SPA and perceived age-based discrimination in the healthcare system. Our findings show that in addition, to sex, financial status and chronic illness, SPA and age-based discrimination in the healthcare were significant predictors of worries and explained additional 8% of the variance. The findings point to the potentially negative impact of the ageism pandemic in an area that has not received much attention, thus far, namely people’s worries. Interventions that address ageism directed by self or others might alleviate worries in the midst of the COVID-19 pandemic.

## 1. Introduction

Worries associated with COVID-19 health consequences are well-justified, as the COVID-19 outbreak was declared a pandemic on March, 11^th^, 2020, by the World Health Organization in the light of its fast spread and lethality [1]. These worries have guided to some extent most of the world leaders to take at least preliminary measures, such as lockdown or physical isolation orders [2]. Similarly, worries concerning the health consequences of the COVID-19 outbreak at the individual level, can be quite adaptive. Those people who report higher levels of worries have shown to follow COVID-19 safety behaviors and avoid social gatherings, increase handwashing and wear face masks, among other things [3, 4]. However, if worries become too intense, then we might see full-blown anxiety, severe mental distress and paralysis that impair people’s functioning [5, 6].

The present study aimed to assess worries concerning COVID-19 health consequences in a nationally representative sample of Israelis over the age of 50. Our main interest was to examine the role of ageism, both directed towards one-self, in the form of self-perceptions of aging (SPA) and perceived to be directed by others based on one’s age. This is because, in addition to its health and mortality impact, the pandemic has a major psychological and social impact [7, 8]. With regard to older adults, ageism, defined as stereotypes, prejudice and discrimination based on age, has been noted as a major side effect spreading at least as fast as the virus [9–11]. Specifically, views of older adults as vulnerable or a burden to society have been widespread, with terms such as “BoomerRemover” reflecting societal dismay towards older adults and a growing intergenerational tension [12].

SPA represent people’s attitudes, beliefs and stereotypes towards their own aging process. As SPA are largely affected by age stereotypes, including societal views of aging [13], which have been at the forefront during the current pandemic [9–11, 14–16], we examined the potential role of SPA in worries about COVID-19 health consequences. Although to date, SPA have not been examined in relation to COVID-19 health worries, it is expected that more negative SPA would be associated with more worries, especially given the societal emphasis on older adults as an at-risk group during the pandemic. Even though chronological age is an independent risk for COVID-19 mortality, we argue that the interpretation of one’s aging process as measured by SPA is likely an important predictor of COVID-19 health worries.

Another variable that has received substantial attention in relation to COVID-19 is age-based discrimination [9–11]. Varied COVID-19 policies and measures represent either a commission or an omission solely based on one’s chronological age [17]. For instance, some countries enforced an age-based criterion for lockdown and social isolation orders, whereas others used such a criterion for their exit strategy [10]. In the healthcare system, an age-based criterion has received the most attention, given fears about the collapse of the healthcare system [9, 18]. In several countries, chronological age was employed as a criterion for the provision of health services or for access to the intensive care unit [19]. In this context, perceived age-based discrimination in the healthcare system may play a major role in people’s worries about COVID-19 health consequences.

Against these two important predictors, we examined demographic characteristics and objective known-risks associated with COVID-19 mortality. Specifically, much of the literature has focused on age and sex as demographic variables of potential association with COVID-19 health worries [3, 20]. Indeed, chronological age is a well-known risk for COVID-19 associated death and severe medical complications [21]. Similarly, men also constitute a risk group [21]. Nevertheless, research concerning the association between age, sex and COVID-19 worries has been somewhat mixed. One study has found that older men were particularly unworried about COVID-19 [3]. A nationally representative study conducted in the United States showed that older adults were more concerned about the mortality risk of COVID-19, but less concerned about getting COVID-19, getting quarantined or being financially impacted by the pandemic [20].

In addition to age and sex, chronic medical conditions and obesity have received a substantial attention as major risk factors for COVID-19 associated death and complicated illness [22, 23]. Health behaviors such as physical activity or smoking [24] also are potential risk factors for COVID-19 associated death, with physical activity being a possible protective factor associated with improved functions [25, 26]. These risk and protective factors have received limited consideration in relation to COVID-19 worries, thus far, though one would expect increased worries among those individuals who are at a greater risk for COVID-19 death and complicated illness [3, 20].

Using a comprehensive perspective to conceptualize COVID-19 related worries, this study has focused on SPA and perceived ageism as two important factors, possibly related to individuals’ ability to emotionally cope with the current pandemic, as manifested in their health-related worries. As ageism (both directed towards one-self and directed by others) has been identified as a major outcome of the current pandemic [9–11], we hypothesized that people’s SPA and perceived discrimination based on age play a major role in people’s COVID-19 worries. Thus, we point to additional negative outcomes of the current pandemic and its impact on older individuals.

## 2. Methods

### 2.1. Participants and procedures

The study was ethically approved by Bar Ilan University. Data are based on a nationally representative survey of 1,092 adults aged 50 and above in Israel. Surveys were conducted in Hebrew via the telephone by trained interviewers between March 29 2020 and May 3 2020. The sample for the current study consisted of adults who had full information on all the study variables. Thus, it consisted of 976 participants.

We carried out selectivity analysis by comparing adults who had full information to those who had missing information. Participants who did not have full information had higher SPA and reported higher age-based discrimination. Participants did not differ in terms of worries, age, gender, financial status, number of chronic illnesses, BMI, physical activity and smoking.

### 2.2. Measures

#### 2.2.1. Health worries of COVID-19

Participants were asked to rate the extent to which they were worried about their health following the coronavirus outbreak. Responses were rated on a scale from 0 ("not at all") to 10 ("to a great extent"), with higher scores representing more COVID-19 related health worries. This measure was selected for ease of use over the phone.

#### 2.2.2. Self-perceptions of aging

Adults’ perceptions of their aging were assessed using the 5-items "Attitudes toward aging" subscale of the "Philadelphia Geriatric Center Morale Scale" [27]. This scale assesses participants’ evaluation of their experiences of aging. Each item has response options ranging from "Strongly disagree" (1) to "Strongly agree" (6) and a mean score was calculated. The scale had good internal reliability in the current sample (α = 0.75).

#### 2.2.3. Age discrimination in healthcare

We also measured experiences of discrimination in the healthcare system (i.e. during visits to doctors, HMOs or hospitals). Respondents were asked how often they had four discriminatory experiences in the healthcare system because of their age (e.g. "being treated with less respect because of your age"). These options were based on the leave-behind questionnaire of the Health and Retirement Survey [28]. We calculated a dummy variable that received 1 if participants reported having any such experience, and 0 if they reported no such experiences.

#### 2.2.4. Social-demographics

Background information was gathered using age as a continuous variable and gender as dichotomous. Financial status was obtained by asking respondents to rate the extent to which their household can make ends meet financially, with response options ranging from 1 ("With great difficulty") to 4 ("Easily").

#### 2.2.5. Health

We examined four chronic illnesses that have been related to elevated COVID-19 risks: diabetes, high blood pressure, heart problems and arthritis. These variables were dummy coded to indicate the existence or absence of each illness. These illnesses were summed to a variable ranging from 0 to 4 chronic illnesses. We also used a measure of BMI (Body Mass Index).

#### 2.2.6. Health behaviors

Smoking was a dummy variable which indicated whether the respondent currently smoked. Physical activity indicated the frequency of engaging in moderate or vigorous physical activity, with response options ranging from 1 ("5–7 times a week") to 5 ("Almost never or never"). The responses were recoded such that a higher score indicated more frequent engagement in physical activity.

### 2.3. Statistical analyses

We began our analyses with descriptive data of the study sample. We then conducted bivariate analyses of the study variables with the dependent variable of health worries of COVID-19. For continuous variables, we used Pearson correlations to assess bivariate associations. To assess the associations between dichotomous and continuous variables, we relied on t-tests. The main stage of analysis was a hierarchical regression model that predicted the dependent variable. The first model included only the socio-demographic variables. The second model added the chronic illnesses and BMI. The third model added health behaviors and the fourth model added SPA and experiences of discrimination in healthcare. We checked for multicollinearity by computing the Variance Inflation Factor (VIF). In our analysis none of the VIF scores exceeded the value of 2, indicating no multicollinearity.

## 3. Results

### 3.1. Demographic and clinical characteristics

Table 1 shows the sample characteristics of the study. Participants reported a medium level of worries, indicated by an average score of 5 out of 10. The sample mean age was 64 and almost half were women. They rated their financial status as medium, and similarly rated their health. Participants reported relatively positive SPA. Over a fifth experienced discrimination due to their age in the healthcare system. The sample had less than one chronic illness on average. They reported medium engagement in physical activity and 14% smoked.

**Table 1:** Sample characteristics and bivariate analyses.

Variable	Mean (SD)	%	Range	Bivariate analyses
Worries	4.81 (3.17)		0–10	r = 1.00***
Age	63.54 (9.21)		50–91	r = 0.01
Sex: women		47.54%		t = -5.67***
Financial status	2.60 (0.96)		1–4	r = -0.20***
Chronic illnesses	0.69 (0.90)		0–4	r = 0.13***
BMI	27.23 (4.74)		16–62	r = 0.05
Physical activity	3.46 (1.39)		1–5	r = -0.08**
Smoking		13.93%		t = -0.01
	0.69 (0.90)		0–4	r = 0.13***
Discrimination in healthcare		22.23%		t = -6.52***
Self-perceptions of aging	4.24 (1.22)		1–6	r = -0.34***

Table 1 also shows bivariate associations with the outcome variable. Individuals who were worried about their health following the COVID-19 outbreak were more likely to be women, had worse financial status, had more chronic illnesses and engaged less frequently in physical activity, had worse SPA and were more likely to experience age-based discrimination in healthcare.

The main stage of the analysis was regressing health worries related to COVID-19 on the independent study variables, controlling for sociodemographic variables and risk factors (Table 2). The first model included the socio-demographic variables and showed that those who were worried were also more likely to be women and in a worse financial status. Age was not related to health worries. The second model added the chronic illnesses and BMI. In addition to the associations with the socio-demographic variables, this model showed that people who had more chronic illnesses tended to be worried about their health during the COVID-19 outbreak. It added two percent to the explained variance. The third model inserted the health behaviors, showing that physical activity and smoking were not related to health worries when controlling for background variables. The fourth and final model added SPA and age-based discrimination in healthcare, and showed that these variables were related to more worries and explained an additional eight percent of the variance in COVID-19 health worries. That is, people who perceived their aging as worse and experienced age-based discrimination in healthcare settings were more likely to report worries about the COVID-19 health consequences.

**Table 2:** Hierarchical linear regression models of worries about health in relation to COVID-19.

	Model 1		Model 2		Model 3		Model 4	
Variable	B (SE)	*p*	B (SE)	*p*	B (SE)	*p*	B (SE)	*p*
*Socio-demographics*:								
Age	0.02 (0.01)	0.133	0.00 (0.01)	0.970	0.00 (0.01)	0.935	-0.01 (0.01)	0.423
Sex: women	1.07 (0.20)	**0.000**	1.24 (0.20)	**0.000**	1.23 (0.20)	**0.000**	1.15 (0.19)	**0.000**
Financial status	-0.63 (0.10)	**0.000**	-0.56 (0.10)	**0.000**	-0.55 (0.10)	**0.000**	-0.31 (0.10)	**0.003**
*Health*:								
Chronic illnesses			0.51 (0.12)	**0.000**	0.50 (0.12)	**0.000**	0.33 (0.12)	**0.004**
BMI			0.03 (0.02)	0.168	0.02 (0.02)	0.269	0.02 (0.02)	0.327
*Health behaviors*:								
Physical activity					-0.10 (0.07)	0.185	0.05 (0.07)	0.467
Smoking					-0.07 (0.29)	0.798	0.01 (0.28)	0.964
*Ageism*:								
Discrimination in healthcare							0.57 (0.24)	**0.019**
Self-perceptions of aging							-0.71 (0.09)	**0.000**
R^2^		0.07		0.09		0.09		0.17

*Note*. Significant p values are in bold.

## 4. Discussion

The present study aimed to assess worries concerning COVID-19 health related consequences in a national sample of Israelis over the age of 50. SPA and perceived age-based discrimination in the healthcare system were selected in the light of the growing presence of ageism during the pandemic [3, 20]. Indeed, the main finding concerns the role of ageism, both directed towards oneself and experienced by others in determining COVID-19 related health worries. Moreover, ageism was found to explain more of the variance compared to objective risk factors.

Our findings show that indeed, both SPA and perceived age-based discrimination in the healthcare system were significant predictors of worries concerning COVID-19 health consequences. As both ageist stereotypes and practices have received an increasing presence during the pandemic [10, 12, 29], it appears that those most affected by ageism also are likely to experience substantial worries during the pandemic. Hence, we point to an unexplored effect of ageism on those 50 and over during the current pandemic.

Age was not a significant correlate of worries concerning COVID-19 health consequences. This finding is unexpected in the light of the fact that it is a well-known risk factors for COVID-19 [30]. The finding also is unexpected in the light of a recent study that has shown that older persons more so than younger persons report high levels of worry concerning the COVID-19 health consequences [20]. This shows how one’s SPA and perceived age discrimination possibly play a greater role than one’s chronological age. Health, however, was associated with worries concerning COVID-19 health consequences. This finding is expected as health complications are a known risk for COVID-19 mortality and severe illness [21, 31].

Two sociodemographic variables were found to be correlated with worries in the present study. Specifically, women were more likely to worry than men and individuals of poorer financial status reported more COVID-19 health worries. The association between sex and worries was in the opposite direction of the heightened risk for men [32]. This is consistent with past research, which has found that older men report fewer worries than women [3]. Financial status, on the other hand operated in the same way as the actual risk for COVID-19 [33], with poorer financial status being associated with higher levels of worry.

Despite its strengths, this study has several limitations. First, the study was conducted in a single country and results may not be generalizable to other countries. In addition, the representativeness of the current sample is limited to Israeli Jews. Future research will benefit from examining the findings in relation to minority groups. It also is important to note that we did not control for various health variables such as cognitive functioning or disability. Finally, it is important to note that we relied on different measures to assess the hypothetical constructs of self- and other-directed ageism. These measures likely are constrained in their scope and do not concern all contexts in which self-and other-directed ageism exists. The measures also did not assess positive aspects of ageism, which also were present during the pandemic [34].

### 4.1. Conclusions and implications

Nevertheless, the findings point to the important role of ageism in people’s worries. People’s SPA and perceived discrimination based on age were shown to be significant predictors of worries concerning COVID-19 health consequences. This points to the potentially negative impact of the ageism pandemic in ways that have not received attention, thus far. As ageism is one of the unintended consequences of the current pandemic, it is likely that people’s ability to cope with the pandemic are directly linked with current affairs, thus potentially exacerbated by ageist messages and policies. Interventions that address ageism both at the individual level and at the societal level can potentially alleviate individuals’ worries concerning COVID-19 health consequences and thus, improve people’s ability to cope with the current pandemic.
